# Increasing the Efficacy of Treatment of *Staphylococcus aureus*–*Candida albicans* Mixed Infections with Myrtenol

**DOI:** 10.3390/antibiotics11121743

**Published:** 2022-12-02

**Authors:** Ruba Y. Mahmoud, Elena Y. Trizna, Rand K. Sulaiman, Roman S. Pavelyev, Ilmir R. Gilfanov, Svetlana A. Lisovskaya, Olga V. Ostolopovskaya, Larisa L. Frolova, Alexander V. Kutchin, Galina B. Guseva, Elena V. Antina, Mikhail B. Berezin, Liliya E. Nikitina, Airat R. Kayumov

**Affiliations:** 1 Institute of Fundamental Medicine and Biology, Kazan Federal University, 420008 Kazan, Russia; 2 Varnishes and Paints Department, Kazan National Research Technological University, 420015 Kazan, Russia; 3 Faculty of Medicine and Biology, Kazan State Medical University, 420012 Kazan, Russia; 4 Scientific Research Institute of Epidemiology and Microbiology, 420015 Kazan, Russia; 5 Institute of Chemistry, Federal Research Center “Komi Scientific Centre”, Ural Branch, Russian Academy of Sciences, 167000 Syktyvkar, Russia; 6 G.A. Krestov Institute of Solution Chemistry of Russian Academy of Sciences, 153045 Ivanovo, Russia

**Keywords:** mixed infections, myrtenol, benzalkonium chloride, drug synergism, *Staphylcoccus aureus*, *Candida albicans*

## Abstract

Infectious diseases caused by various nosocomial microorganisms affect worldwide both immunocompromised and relatively healthy persons. Bacteria and fungi have different tools to evade antimicrobials, such as hydrolysis damaging the drug, efflux systems, and the formation of biofilm that significantly complicates the treatment of the infection. Here, we show that myrtenol potentiates the antimicrobial and biofilm-preventing activity of conventional drugs against *S. aureus* and *C. albicans* mono- and dual-species cultures. In our study, the two optical isomers, (−)-myrtenol and (+)-myrtenol, have been tested as either antibacterials, antifungals, or enhancers of conventional drugs. (+)-Myrtenol demonstrated a synergistic effect with amikacin, fluconazole, and benzalkonium chloride on 64–81% of the clinical isolates of *S. aureus* and *C. albicans*, including MRSA and fluconazole-resistant fungi, while (−)-myrtenol increased the properties of amikacin and fluconazole to repress biofilm formation in half of the *S. aureus* and *C. albicans* isolates. Furthermore, myrtenol was able to potentiate benzalkonium chloride up to sixteen-fold against planktonic cells in an *S. aureus*–*C. albicans* mixed culture and repressed the adhesion of *S. aureus*. The mechanism of both (−)-myrtenol and (+)-myrtenol synergy with conventional drugs was apparently driven by membrane damage since the treatment with both terpenes led to a significant drop in membrane potential similar to the action of benzalkonium chloride. Thus, due to the low toxicity of myrtenol, it seems to be a promising agent to increase the efficiency of the treatment of infections caused by bacteria and be fungi of the genus Candida as well as mixed fungal–bacterial infections, including resistant strains.

## 1. Introduction

Infectious diseases caused by various nosocomial bacteria and fungi like Enterobacteriaceae (*Klebsiella* sp. and *Escherichia coli*), *Staphylococcus aureus*, *Candida albicans, Cryptococcus neoformans*, and many others affect worldwide both immunocompromised and relatively healthy persons [1]. In addition to the most vulnerable populations of patients, such as neonatal, old, and AIDS-infected patients and persons with an intravenous catheter, in the last three years, SARS-CoV2 led to an increased risk of mortality and a longer course of ICU stays [2,3,4]. Antimicrobial therapy remains the only way to target pathogenic microorganisms and save lives. Although conventional antimicrobial agents use various strategies to repress the growth of pathogens, bacteria and fungi have different tools to evade them, making the development and spread of antimicrobial resistance (AMR) one of the factors that complicates the treatment of infectious diseases [5]. It has been shown in the last decades that, in many cases, several pathogens rather than only one are associated with disease [6]. These polymicrobial infections are often characterized by more intense symptoms than any of the effects noticed by one microbe alone and increased resistance to treatment [7,8]. *S. aureus* and *C. albicans*, an important, dangerous twosome, have been shown to form a bacterial–fungal environment and were coisolated from different infections, including periodontitis, cystic fibrosis, denture stomatitis, urinary tract infections, burn wound infections, and infections of medical devices, such as central venous catheters [9]. In bacterial–fungal coinfection, each counterpart has been reported to contribute to resistance [10,11,12]. Moreover, 94% of *S. aureus* isolates are tolerant to penicillin and its derivatives [13], and even cephalosporins and carbapenems often become ineffective against this bacterium, leading to increased mortality of *S. aureus*-associated infections [14]. Some of the resistance mechanisms of *S. aureus* are limiting the drug uptake, modifying the drug target, inactivating the drug, and active drug efflux [15]. *C. albicans* also busts resistance to antifungals along the course of treatment [16] via transforming between several morphological forms (blastospores, pseudohyphae, and hyphae) [17], decreasing the permeability of drugs, and expressing efflux pumps or compromised drug import [18].

In addition, biofilm formation plays an important role in *S. aureus* and *C. albicans* protection. Biofilms are microbial communities (either mono- or polymicrobial) where the cells are embedded into a matrix consisting of polysaccharides, proteins, and nucleotides produced by the cells themselves [9,19,20]. The biofilm is formed in several stages, including attachment to biotic or abiotic surfaces, maturation, and detachment (dispersal of mature biofilm) [21]. While in biofilm, microorganisms are characterized by a decreased susceptibility to antimicrobials due to the diffusional barrier for the latter as well as being more virulent and capable to adhere to surfaces and form new biofilms [22]. Therefore, the development of new approaches to increase the susceptibility of pathogenic microorganisms to conventional antimicrobials could be promising in overcoming the AMR problem. 

Various classes of compounds were reported to be able to potentiate the efficiency of antimicrobials against planktonic- and biofilm-embedded bacteria and fungi: derivatives of 5(*H*)furanone [23,24], various hydrolytic enzymes [25,26], and essential oils [27,28]. Terpenes, the active fraction of essential oils from plant extracts, make up the largest group of secondary metabolites of plants (over 50,000 known substances) [29]. Monoterpenes consist of two isoprene units and naturally occur in plants and essential oils [30] and are introduced as key ingredients in the design and production of novel biologically active compounds because of anti-inflammatory, antimicrobial, anticonvulsant, analgesic, antiviral, anticancer, antituberculosis, and antioxidant biological activities [31,32,33]. Additionally, some researchers have described the ability of terpenes to inhibit the formation of *S. aureus* biofilms as well as their antimicrobial and antifungal activity [34,35]. Myrtenol is a monoterpene bicyclic derivative that has been well known for its antimicrobial activity [36]. Myrtenol exhibited antibacterial activity against *S. aureus* and *Acinetobacter baumannii* [37,38] and has repressed the growth of *C. albicans*, *R. nigricans*, *A. fumigates*, and *F. solani* fungi species [36]. Several chemically synthesized myrtenol derivatives demonstrated significant in vitro antifungal activity against *Physalospora piricola* with better or comparable antifungal activity than those of positive controls (the commercial fungicides azoxystrobin and chlorothalonil) [32]. In addition, the combination of myrtenol and antifungal agents reduced the effective concentrations of the latter with synergistic and additive effects [39,40]. The mechanism of myrtenol action is discussible. It has been suggested that myrtenol possibly damages the fungal membrane, affecting the change in the functional state of integrin-like proteins, which can lead to the disruption of morphogenesis of the fungal cell [36]. 

Here, we show that myrtenol potentiates the antimicrobial and biofilm-preventing activity of conventional drugs against *S. aureus* and *C. albicans* mono- and dual-species cultures. 

## 2. Results

### 2.1. Antibacterial and Antifungal Activity of Myrtenol

The antimicrobial activity of myrtenol was evaluated on *S. aureus* ATCC 29213 as well as four methicillin-sensitive clinical isolates of *S. aureus* (MSSA), seven methicillin-resistant isolates of *S. aureus* (MRSA), and 10 clinical isolates of *C. albicans*. (−)-myrtenol and (+)-myrtenol exhibited low both antibacterial and antifungal activities (Table 1 and Table 2). Worth noting, the minimum bactericidal concentration (MBC) either fit or exceeded the minimum inhibiting concentration (MIC) two-fold, suggesting the bactericidal/fungicidal property of terpenes. Furthermore, MRSA and MSSA were of similar susceptibility to myrtenol, and the resistance to fluconazole did not affect the susceptibility of *C. albicans* isolates to terpene. 

### 2.2. Myrtenol Potentiates Both Antibacterial and Antifungal Agents

The synergism of myrtenol with antimicrobials was assessed using the chequerboard approach. For *S. aureus*, the concentrations of amikacin or benzalkonium chloride were in the range of 0.06–4 × MIC, and myrtenol was added to concentrations of 0.125–1 × MIC. After 24 h of incubation, the fractional inhibitory concentration index (FICI) was calculated for both the growth and biofilm repression assessed by crystal violet staining (Supplementary Tables S1–S4). (−)-Myrtenol exhibited a synergistic effect with amikacin with an FIC index in the range of 0.3–0.5 on 42% of clinical isolates of *S. aureus*; (+)-myrtenol led to a four-fold decrease of the MIC of antibiotics against 75% of the studied isolates regardless of their susceptibility to methicillin (MRSA or MSSA). On the other isolates, the combined use of amikacin and either (−)- myrtenol or (+)-myrtenol led to an additive effect (Table 3). On the biofilm-preventing activity, the synergistic effect of (−)-myrtenol and (+)-myrtenol with amikacin was observed for 42% and 33% of isolates, respectively (Table 3). 

In a combination of myrtenol with benzalkonium chloride, in most cases, the additive effect was observed against the planktonic cells of *S. aureus* isolates. (−)-Myrtenol potentiated the antiseptic only against one strain, and (+)-myrtenol demonstrated synergy with benzalkonium chloride against four isolates (33%). Regarding biofilm prevention, (−)-myrtenol significantly increased the efficiency of benzalkonium chloride against 50% of isolates with an FICI ranging from 0.16–0.38 while (+)-myrtenol significantly increased the effectiveness of the antiseptic in only 17% of isolates.

Next, the synergistic effect of myrtenol with fluconazole and benzalkonium chloride against *C. albicans* was evaluated. The fractional inhibitory concentration index (FICI) was calculated for planktonic cell growth repression and biofilm formation prevention (Supplementary Tables S5–S8). (+)-Myrtenol exhibited synergy with fluconazole in 64% of the *C. albicans* isolates while (−)-myrtenol mainly led to an additive effect, and synergy was only observed in 36% of isolates (Table 4). By contrast, when assessing the biofilm repression, (−)-myrtenol had an FICI less or equal to 0.5 for six out of 11 isolates while (+)-myrtenol was only for four out of 11 (Table 4). (−)-Myrtenol also demonstrated synergism with benzalkonium chloride in relation to planktonic cells for five isolates, and the combined use of (+)-myrtenol with antiseptic showed a clear synergistic effect in relation to nine isolates. A similar result was obtained for *C. albicans* biofilm repression. Most of the isolates (seven out of 11) were more sensitive to the combination of antiseptic with (+)-myrtenol while the use of (−)-myrtenol with benzalkonium chloride showed an FICI less or equal to 0.5 for only four isolates (Table 4).

Thus, these data indicate that myrtenol reduces the effective concentrations of antimicrobial and antifungal drugs, which, in turn, reduces both the general toxic effect on the host organism and the risk of resistance development by bacterial and fungal cells. 

### 2.3. Myrtenol Increases the Antimicrobial and Antifungal Activity of Benzalkonium Chloride against an S. aureus and C. albicans Mixed Culture

**Figure 1:**
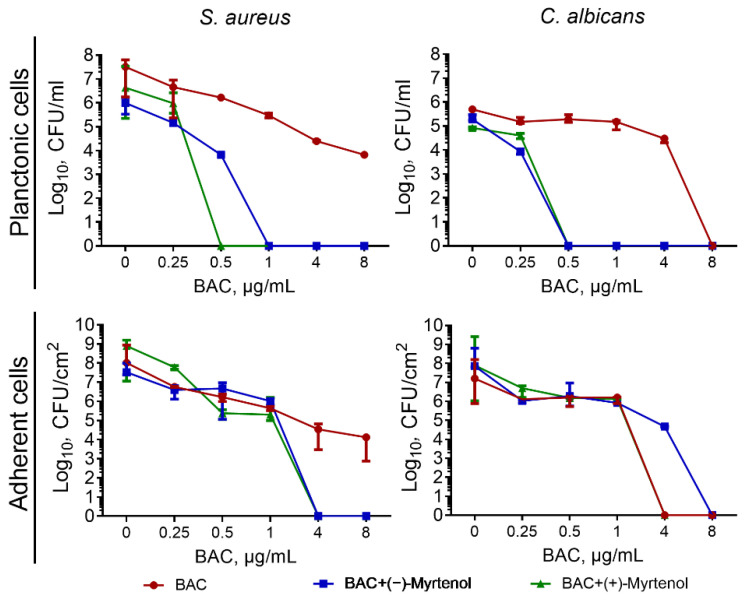
Viability of *S. aureus* and *C. albicans* in mixed culture in presence of benzalkonium chloride with concentrations 0, 0.25, 0.5, 1, 4, and 8 µg/mL separately and in combination with (−)-myrtenol and (+)-myrtenol at a concentration of 256 µg/mL. The viability of bacterial and fungal cells was assessed after 24 h growth in culture liquid and after 48 for adherent cells. The viable cells were counted after a series of ten-fold dilutions followed by plating on selective media for differentiation of *S. aureus* and *C. albicans*.

Since the benzalkonium chloride demonstrated synergy with myrtenol against both *S. aureus* and *C. albicans*, the effect of their combined use against the fungal–bacterial mixed culture community was assessed. For this purpose, *S. aureus* and *C. albicans* were cocultivated in a BM broth in a 24-well plate in the presence of benzalkonium chloride in the concentration range of 0, 0.25, 0.5, 1, 4, or 8 µg/mL solely or in combination with (−)-myrtenol and (+)-myrtenol at a concentration of 256 µg/mL. After a 24 h incubation, the viability of planktonic bacterial and fungal cells was assessed by counting CFUs in a series of ten-fold dilutions followed by plating on selective media for the differentiation of *S. aureus* and *C. albicans*. The sole benzalkonium chloride led to a three-log decrease of viable *S. aureus* and the death of *C. albicans* planktonic cells only at 8 µg/mL (Figure 1). In the presence of either myrtenol, (−) or (+), a significant increase in the efficiency of the antiseptic was observed, and the complete death of both *C. albicans* and *S. aureus* planktonic cells was observed at a concentration of 0.5–1 μg/mL, suggesting an eight- to sixteen-fold increase in the antiseptic’s efficiency by terpene. Worth noting, while the combination of both (−)-myrtenol and (+)-myrtenol with benzalkonium chloride led to the prevention of the adherence of *S. aureus*, although at 4 µg/mL of antiseptic, no significant effect on *C. albicans* adherence was observed. 

### 2.4. Myrtenol Damages the Cell Membrane of Bacterial and Fungal Cells

**Figure 2:**
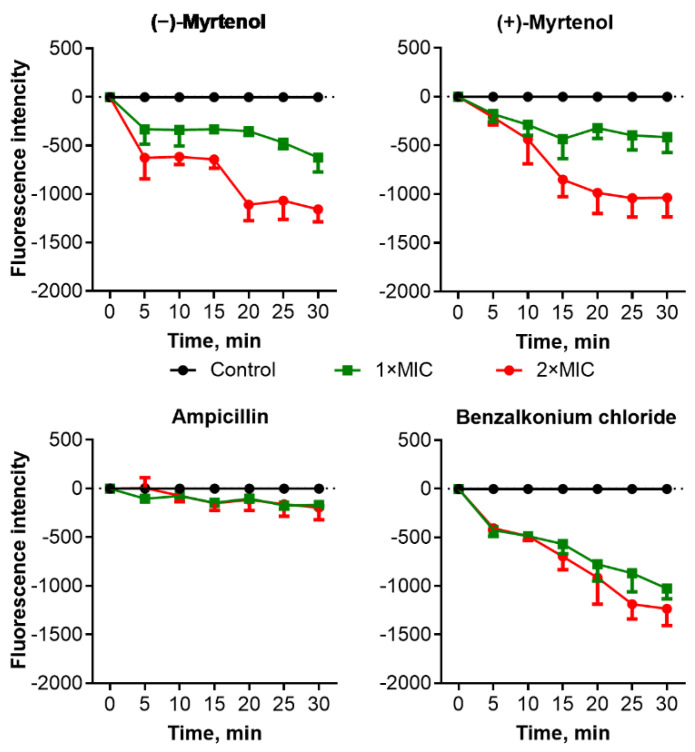
Relative membrane potential of *S. aureus* cells expressed in fluorescence units of DioC2(3) reduced on an intact cell membrane resulting in green fluorescence. Cells were grown for 18 h in LB broth, washed with PBS, and resuspended until a final density of 10^6^ CFU/mL in PBS supplemented with DioC2(3) to a final concentration of 10 μM/mL. After a 30 min incubation at 25 °C, compounds of interest were added to the samples. Fluorescence detection was performed for 30 min with 5 min intervals with excitation and emission wavelengths of 497 and 520 nm, respectively.

**Figure 3:**
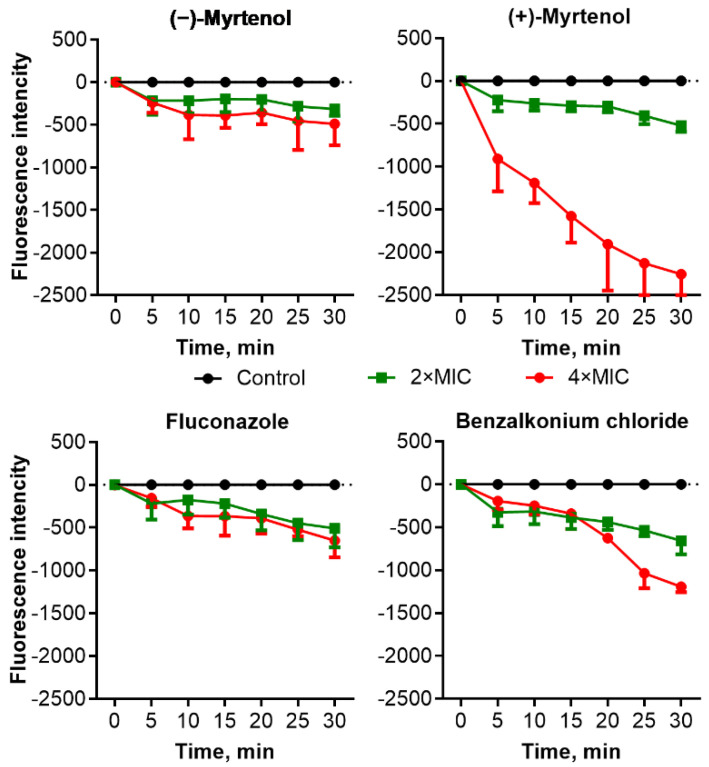
Relative membrane potential of *C. albicans* cells expressed in fluorescence units of DioC2(3) reduced on an intact cell membrane resulting in green fluorescence. Cells were grown for 18 h in Sabouraud broth, washed with PBS, and resuspended until a final density of 10^5^ CFU/mL in PBS supplemented with DioC2(3) to a final concentration of 10 μM/mL. After a 30 min incubation at 25 °C, compounds of interest were added to the samples. Fluorescence detection was performed for 30 min with 5 min intervals with excitation and emission wavelengths of 497 and 520 nm, respectively.

Since damage to the cell membrane has been proposed for various terpenes as the mechanism of antimicrobial action [41], the effect of myrtenol on the membrane potential of bacterial and fungal cells was investigated. Cells were preincubated with the fluorescent dye DioC2(3) which can be reduced on the membrane of intact cells; then the myrtenol was added until 0.5–2×MIC, and the fluorescence was recorded during 30 min of incubation. As can be seen from Figure 2, in the presence of (−)-myrtenol and (+)-myrtenol, the fluorescence intensity of *S. aureus* cells decreased compared to untreated cells in a dose-dependent manner, confirming a drop in membrane potential, apparently, because of its damage. A similar drop in fluorescence was observed in cells treated with benzalkonium chloride, which also permeates the cell membrane, while no changes were detected in ampicillin-treated cells. These data clearly suggest that both (−)-myrtenol and (+)-myrtenol apparently damage the bacterial membrane, thus facilitating the penetration of antimicrobials into the cell. Treatment of *C. albicans* cells with low concentrations of (−)-myrtenol did not affect the fluorescence, similar to fluconazole, although the latter also affects the integrity of the membrane via repression of the conversion of lanosterol to ergosterol (Figure 3). By contrast, (−)-myrtenol led to a significant decrease in fluorescence comparable with the effect of benzalkonium chloride. 

**Figure 4:**
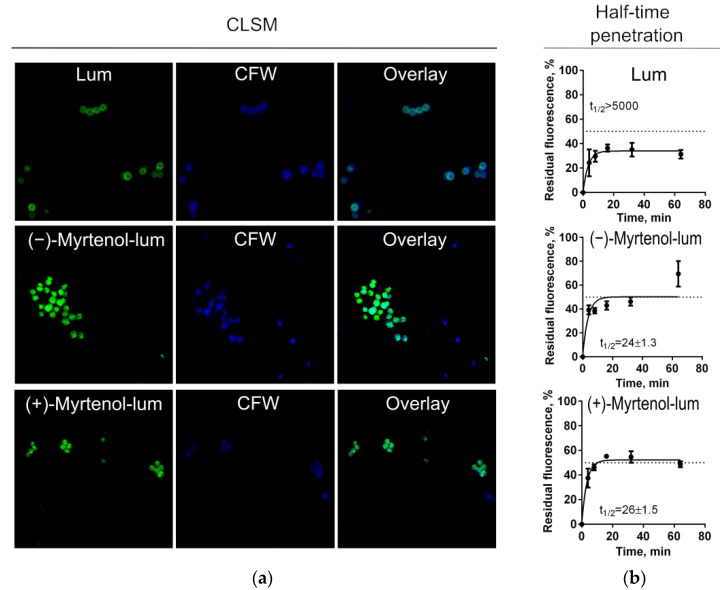
Myrtenol-lum penetration into *S. aureus* cells. (**a**) The localization of either (−)-myrtenol or (+)-myrtenol carrying the fluorophore BODIPY (Myrtenol-lum) assessed by confocal laser scanning microscopy. The solely fluorophore (lum) and Calcofluor-White (CFW) membrane dyes served as references. (**b**) Penetration of myrtenol-lum into *S. aureus* cells. Myrtenol-lum was added to *S. aureus* cells, and the sole fluorophore (lum) was used as a control. After 4, 8, 16, 32, and 64 min of incubation, cells were harvested, washed with PBS, and residual fluorescence was measured. The half-time of penetration (t½) was 24 ± 1.3 min and 26 ± 1.5 min for (−)-myrtenol and (+)-myrtenol, respectively, while for the fluorophore solely (lum) was t½ > 5000 min.

**Figure 5:**
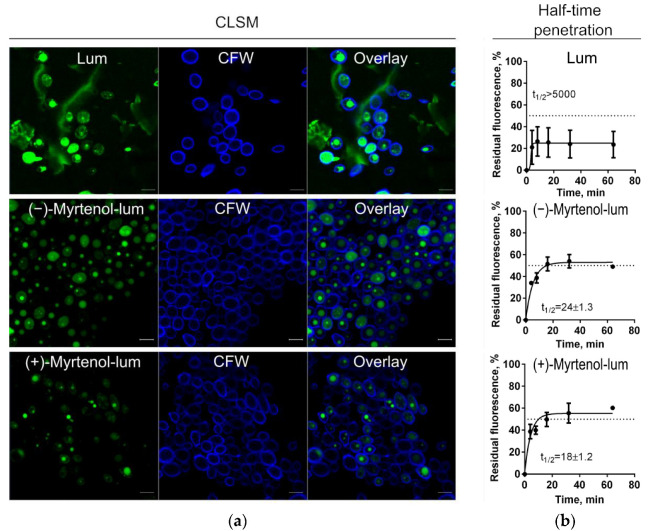
Myrtenol-lum penetration into *C. albicans* cells. (**a**) The localization of either (−)-myrtenol or (+)-myrtenol carrying the fluorophore BODIPY (myrtenol-lum) assessed by confocal laser scanning microscopy. The solely fluorophore (lum) and Calcofluor-White (CFW) membrane dyes served as references. (**b**) Penetration of myrtenol-lum into *C. albicans* cells. Myrtenol-lum was added to *C. albicans* cells, and the sole fluorophore (lum) was used as a control. After 4, 8, 16, 32, and 64 min of incubation, cells were harvested, washed with PBS, and residual fluorescence was measured. The half-time of penetration (t½) was 24 ± 1.3 min and 18 ± 1.2 min for (−)-myrtenol and (+)-myrtenol, respectively, while for the fluorophore solely (lum) was t½ > 5000 min.

To evaluate whether myrtenol binds to the membrane or diffuses into the cell, confocal laser scanning microscopy was performed to check the localization of terpenes in bacterial cells. For this, *S. aureus* and *C. albicans* cells were incubated for 15 min in the presence of myrtenol fused with a fluorophore (myrtenol-lum). Synthesis, physicochemical properties, and spectral data of BF_2_-ms-(4-((1″R)-6″,6″-dimethylbicyclo[3.1.1]hept-2″-ene-2″)ylmethoxycarbonylpropyl)-3,3′,5,5′-tetramethyl-2,2′-dipyrromethene (mentioned as “lum”) were described in detail in our previous paper [42]. To visualize the membranes of bacteria and fungi, cells were additionally stained with CalcoFluor-White (CFW). Figure 4 and Figure 5 show that myrtenol was evenly distributed in *S. aureus* cells (green fluoresce) while the fluorophore was observed only over the cell surface, suggesting that myrtenol diffuses through the membrane. A similar result was shown for *C. albicans* cells (Figure 4 and Figure 5).

In the next step, the rate of penetration of myrtenol into bacterial and fungal cells was assessed. Myrtenol containing a fluorophore in its structure (Myrtenol-lum) was added to *S. aureus* and *C. albicans* cells. The pure fluorophore (lum) itself was used as a control. After 4, 8, 16, 32, and 64 min of incubation, cells were harvested, washed with PBS, and the fluorescence in suspension was measured using a Tecan Infinite 200 Pro microplate reader (Switzerland). Cells without any added compounds were considered point zero, and the cell suspension with the fluorescent compound was considered 100%. As can be seen from Figure 4 and Figure 5, the half-time of maximal penetration (t½) of (+)-myrtenol-lum was 26 ± 1.5 min and 18 ± 1.2 min for *S. aureus* and *C. albicans,* respectively. For (−)-myrtenol-lum, the calculated t½ was 24 ± 1.3 min while the t½ of the sole fluorophore was t½ > 5000 min in both bacterial and fungal cells, suggesting the interaction of myrtenol with the membrane.

## 3. Discussion

The worldwide spread of pathogenic bacteria and micromycetes resistant or tolerant to conventional antimicrobials drastically decreases the number of available options for the treatment of infectious diseases and thus becomes a global challenge for healthcare [43,44,45,46]. Furthermore, the coexistence of different microorganisms in mixed communities leads to additional difficulties in treatment compared to monospecific infections [8,11,47]. Due to interbacterial and bacterial–fungal interactions in consortia, their counterparts change metabolism and morphology that consequently leads to altered susceptibility to antimicrobials [7,48,49]. Therefore, the development of either novel universal antimicrobials or approaches to potentiate conventional ones could be tools to overcome the tolerance of microorganisms to antimicrobials.

Essential oils have been shown as both potential antimicrobials and enhancers of conventional antimicrobials [27,28]. In particular, the bicyclic monoterpene myrtenol, a terpene from the myrtenol tree, is able to repress the growth of bacteria [37,38] and fungi [36] as well as reduce the effective concentrations of some antifungals [39,40]. In our study, the two optical isomers of myrtenol, (−)-myrtenol and (+)-myrtenol, were tested as either antibacterial, antifungal, or enhancers of conventional drugs. As can be seen from Table 3 and Table 4, (+)-myrtenol demonstrated the synergistic effect with amikacin, fluconazole, and benzalkonium chloride on most of the clinical isolates of *S. aureus* and *C. albicans* while (−)-myrtenol exhibited synergy with conventional drugs only on a third of the isolates. Thus, in the presence of myrtenol, the MICs of amikacin, fluconazole, and benzalkonium chloride were reduced up to sixteen-fold (see Supplementary file), reaching medically relevant concentrations. On the contrary, (−)-myrtenol more readily increased the property of amikacin and fluconazole to repress biofilm formation by the *S. aureus* and *C. albicans* isolates, respectively. The reason for such selectivity remains questionable since the half-time penetration of both (−)-myrtenol and (+)-myrtenol into either *S. aureus* or *C. albicans* was similar at 18–24 min (Figure 4 and Figure 5). Additionally, the confocal microscopy of treated cells revealed similar intracellular localization of (−)-myrtenol and (+)-myrtenol fused to the fluorophore. However, in the membrane integrity assay, (+)-myrtenol led to a faster drop in the membrane potential of treated *C. albicans* cells (Figure 3), which allows for speculation about either the specificity of (+)-myrtenol to any molecular target or a higher tropism to the membrane at least in fungal cells. The last assumption may be less probable since no difference in the effect of either (−)-myrtenol or (+)-myrtenol on the *S. aureus* membrane could be observed (Figure 2). Nevertheless, the mechanism of both (−)-myrtenol and (+)-myrtenol synergy with conventional drugs is apparently driven by membrane damage since the treatment with both terpenes led to a drop in membrane potential similar to the action of benzalkonium chloride (Figure 2 and Figure 3), the membrane-permeating agent [50,51,52].

As has been reported in many works, *S. aureus* and *C. albicans* are opportunistic pathogens that live in the same niche and are capable of forming mixed-species consortia. These consortia appear widely on various mucosa, including the mouth, vaginal tract, etc. [10]. In this form, their resistance to antimicrobial and antifungal drugs increases significantly [53,54]. Hence, we tested whether either (−)-myrtenol or (+)-myrtenol could potentiate the antiseptic benzalkonium chloride against a mixed culture of *S. aureus* and *C. albicans*. As can be seen from Figure 1, in this case, both isomers of myrtenol were able to potentiate benzalkonium chloride up to sixteen-fold against planktonic cells, which allows for reduction of the concentration of this toxic antiseptic for the treatment of various mucosa with the same efficiency. On the other hand, the increase in antiseptic efficiency decreases the risk of resistance development by pathogens [55]. Unfortunately, while the combination of myrtenol with antiseptic could completely repress the adhesion of *S. aureus*, no effect of terpene on *C. albicans* adhesion repression by benzalkonium chloride could be observed. This effect is probably due to the highly adaptive capabilities of the fungal cells that make it possible to neutralize the negative effect of antimycotics at their low concentrations.

Taken together, our data allow for the suggestion of myrtenol as a tool to increase the susceptibility of pathogens to antimicrobials. While the terpene will apparently not be effective against resistant strains, its combined use with antimicrobials could be helpful when treating tolerant isolates. The lack of toxicity of terpenes [56,57] makes them a harmless and potential therapeutic agent to increase the efficiency of the treatment of bacterial and fungal infections mediated by resistant strains. Thus, in much previous research, neither cytotoxicity nor acute toxicity on animals has been found for relatively high concentrations of myrtenol, up to 600 mg/L in vitro and 1.3 g per kg in vivo [58,59,60]. It is worth mentioning that a crucial benefit from using the described compounds is that their resource is almost inexhaustible [61,62]. Thus, the knowledge of the clinical and economic burden of antibiotic-resistant mixed infections, coupled with the benefits of the availability of such compounds, will allow for optimal control and improved patient safety [63].

## 4. Materials and Methods

### 4.1. Chemistry

The (+)- or (−)-myrtenol were synthesized by the oxidation of (+)- or (−)-α-pinene with tert-butyl hydroperoxide in the presence of catalytic amounts of SeO_2_ according to the reported procedure [64]. The myrtenal formed during the reaction (content 70–75% by Gas liquid chromatography) was isolated through a water-soluble sulfite derivative (aldehyde purity is 97–98%) with subsequent NaBH_4_ reduction of the aldehyde into myrtenol. A yield of 40–42% was observed. The spectral data and physical constants associated with the compounds obtained fit with the literature data. Synthesis, physicochemical properties, and spectral data of BF_2_-ms-(4-((1″R)-6″,6″-dimethylbicyclo[3.1.1]hept-2″-ene-2″)ylmethoxycarbonylpropyl)-3,3′,5,5′-tetramethyl-2,2′-dipyrromethene (mentioned as “lum”) were described in detail in our previous paper [42]. A solution of ester 1 (0.128 mmol, 1 equiv) in isopropanol (5 mL) was stirred with 0.1 N KOH (2 mL) under argon atmosphere at room temperature using thin layer chromatography (TLC) in a 1:10 methyl tert-butyl ether (MTBE)−CCl_4_ system to monitor the reaction progress. After almost complete transformation (1−2 h), the mixture was evaporated. Then, 20 mL of toluene and diluted aqueous HCl were added to the mixture with intensive stirring. The organic layer was separated and evaporated in vacuo. Then, 0.154 mmol (1.2 equiv) of (−)- or (+)-myrtenol and 0.128 mmol of DMAP in 20 mL of dichloromethane (DCM) were added. After complete dissolution, 0.384 mmol of HATU was added to the mixture. The progress of the reaction was monitored by TLC with a 1:10 MTBE−CCl4 system. After completion of the reaction (about 5 h), the solvent was removed in vacuum and the product was purified by silica gel column chromatography. A 1:19 MTBE−CCl_4_ mixture served as an eluent. A yield of 59% was observed. The stock solutions of (−)-myrtenol and (+)-myrtenol were prepared in pure DMSO at a concentration of 20 g/L. Working solutions were prepared in a bacterial growth medium with a final concentration of DMSO of no more than 5%, which is nontoxic for both bacterial and fungal strains. Amikacin (Sigma, Rehovot, Israel), benzalkonium chloride (Sigma), and fluconazole (Sigma) were used as reference antimicrobials.

### 4.2. Strains and Cultivation Conditions

A methicillin-sensitive *Staphylococcus aureus* ATCC 29213 as well as 10 clinical MRSA isolates obtained from the Republican Clinical Hospital, Laboratory of Clinical Bacteriology in Kazan were used in this study (see Table 1). The bacterial strains were stored in 50% (V/V) glycerol stocks at −80 °C and freshly streaked on LB plates followed by their overnight growth at 37 °C before use. Ten clinical isolates of *Candida albicans* (see Table 2 for resistance details) from the patients of Kazan Scientific Research Institute of Epidemiology and Microbiology (Kazan, Russia) obtained during the year 2019 were used. Isolates were identified as *C. albicans* by using AuxaColor 2 Colorimetric sugar-assimilation yeast-identification kit (Bio-Rad) and confirmed on MALDI-TOF mass spectrometry (Bruker Biotyper system, Bruker Daltonics, Germany). Fungal strains were stored as a 50% glycerol stock at −80 °C and grown in the RPMI broth. The overnight cultures were used to adjust an optical density to 0.5 McFarland (equivalent to 10^8^ cells/mL) in growth medium and used as a working suspension. To obtain a mature biofilm, fungal and bacterial cells were seeded in TC-treated culture plates (at 10^6^ cells/mL) and grown under static conditions for 48 h at 37 °C in BM-broth [65] supplemented with 1% glucose. Mannitol salt agar and Sabouraud agar supplemented with ciprofloxacin (20 μg/mL) were used for the differential count of CFUs of *S. aureus* and *C. albicans*, respectively, in *S. aureus*–*C. albicans* mixed cultures.

### 4.3. Determination of the Minimum Inhibitory (MIC) and the Minimum Bactericidal/Fungicidal Concentrations (MBC/MFC)

The minimum inhibitory concentration (MIC) was determined by serial microdilution in 96-well plates according to the EUCAST rules for antimicrobial susceptibility testing [66]. The highest final concentration of each compound was 512 µg/mL. The next wells contained two-fold decreasing concentrations of compound in the range of 0.5–1024 μg/mL. The wells were seeded with microbial culture to obtain density of 106 CFU/mL in a volume of 200 μL per well. The plates were incubated under static conditions at 37 °C for 24 in case of bacterial culture and 48 h for yeast. The growth was assessed by measuring the optical density at wavelength of 600 nm. The minimum inhibitory concentration of the compound was defined as the concentration providing complete suppression of the visible growth of cells. The minimum bactericidal/fungicidal concentration (MBC/MFC) was determined by seeding 5 μL of culture fluid from wells with no visible growth in 3 mL of fresh nutrient broth. The MBC/MFC was considered the minimum concentration of the studied compound, which ensures the complete absence of growth [67].

### 4.4. Determination of the Biofilm Prevention Concentration (BPC)

To determine the biofilm prevention concentration (BPC), bacterial and fungal cells were grown in 96-well adhesive plates for 48 h under static conditions at 37 °C in BM broth in wells of 200 μL with an initial density of 106 CFUs / ml in the presence of the test substances. Next, staining with crystal violet was carried out as described in [68]. The minimum biofilm inhibitory concentration was defined as the lowest concentration providing no visible staining of the residual biofilm.

### 4.5. Analysis of the Antimicrobial Effect in the Combined Use of Antimicrobial Agents (Chequerboard Approach)

The chequerboard approach was used to assess the possibility of increasing the effectiveness of other antimicrobial agents with myrtenol. The experimental methodology was similar to the determination of the MIC in 96-well plates. Each plate contained serial dilutions of a myrtenol derivative and various compounds in a chequerboard pattern, as described previously [69]. One of the antimicrobial substances [A] was twice diluted horizontally, and the other [B] vertically on a 96-well plate. The result was a combination of 77 concentrations of antimicrobial compounds [A] and [B]. The extreme lines and columns contained only one of the considered substances to determine their MICs directly in the experiment. The initial concentration of each studied antimicrobial agent was 4 × MIC. The final concentration of bacterial and fungal cells in the wells was 0.5 × 10^5^ CFU/mL. The plates were incubated at 37 °C for 20 h. Then, the optical density OD600 was measured on an Infinite 200 PRO plate spectrophotometer (Tecan, Switzerland). Each test was run in triplicate and included a growth control without the addition of any antimicrobial agent. The fractional inhibitory concentration index (FICI) for each double combination was calculated as follows:

(1) $$\mathrm{FICI}=\frac{\mathrm{MIC}\left[A\right]\;\mathrm{in}\;\mathrm{combination}}{\mathrm{MIC}\;\left[A\right]}+\frac{\mathrm{MIC}\left[B\right]\;\mathrm{in}\;\mathrm{combination}}{\mathrm{MIC}\left[B\right]}$$

Interpretation of the obtained FICI values was carried out according to [70,71]; FICI ≤ 0.5 corresponded to synergy, 0.5 < IFIC ≤ 4 to an additive effect while IFIC > 4 corresponded to antagonism.

### 4.6. Evaluation of Viability of Bacterial and Fungal Cells

To assess the viability of planktonic cells, samples from the upper layer of the culture liquid were taken. Then the culture liquid was removed from the wells; wells were washed several times with a sterile NaCl solution (0.9%) to remove planktonic and detached cells. The biofilms were mechanically destroyed, and cells were resuspended in a sterile NaCl solution (0.9%). The viability of cells was evaluated by the drop plate assay with minor modifications [72]. Serial ten-fold dilutions from each well were prepared, and 5 µL of suspension was dropped on Mannitol salt agar and Sabouraud agar with ciprofloxacin (20 μg/mL) to differentiate *S. aureus* and *C. albicans* cells, respectively. After 24 h of incubation at 37 °C, the number of colonies on the plates was counted; the values were averaged and expressed as CFU/mL.

### 4.7. Membrane Potential Evaluation

Membrane potential was evaluated by the detection of 3,3’-diethyloxacarbocyanine iodide (DioC2(3)) fluorescence as an indicator of the membrane potential level. Bacterial or fungal cells were grown for 18 h in LB broth with stirring, then harvested and washed with PBS. Cells were resuspended until a final density of 10^6^ CFU/mL was reached in PBS supplemented with DioC2(3) to a final concentration of 10 μM/mL. *C. albicans* cells were resuspended until a final density of 10^5^ CFU/mL. After a 30 min incubation at 25 °C, compounds were added to the samples. Fluorescence detection was performed for 30 min with 5 min intervals using carboxyfluorescein (FAM) wavelength detection (the excitation and emission wavelengths were 497 and 520 nm, respectively).

### 4.8. Estimation of the Penetration Rate of Myrtenol into Bacterial and Fungal Cells

To assess the penetration rate of terpenoids into bacterial cells, (−)-myrtenol-lum and (+)-myrtenol-lum, which contain a fluorophore (lum) in their structures, were used. Bacterial and fungal cells were grown overnight at 37 °C in LB culture medium with agitation, then washed with BPS (pH = 7.4), and resuspended in a buffer to an optical density of 0.5 by McFarland. Either (−)-myrtenol-lum or (+)-myrtenol-lum was added to a final concentration of 10 µg/mL and incubated at 25 °C in the dark. Pure fluorophore was used as a control. After 4, 8, 16, 32, and 64 min of incubation, 150 µL of the suspension was taken; cells were harvested by centrifugation, washed with PBS, and then resuspended in 150 µL of the buffer. The fluorescence was measured using a Tecan Infinite 200 Pro microplate reader (at an excitation and emission wavelength of 485 and 520 nm, respectively). The time required to obtain half of the maximum fluorescence of stained cells (t 1/2) was calculated by plotting log10 (time) as a function of percent fluorescence (taking into account the fluorescence of unstained cells as 0% and the fluorescence of cell suspension in buffer with test compound (10 µg/mL) as 100%) in GraphPad Prism 6. Additionally, the penetration of either (−)-myrtenol-lum or (+)-myrtenol-lum in bacterial and fungal cells and their localization there were assessed by confocal laser scanning microscopy on microscope. (−)-Myrtenol-lum, (+)-myrtenol-lum, or pure fluorophore were added to the cells at a concentration of 10 μg/mL. Cell membranes were additionally stained with calcofluor dye (1 mg/mL). As a result, the membranes that were stained in blue (excitation emission) and green fluorescence (excitation emission) indicated the localization of terpenes in the cells.

### 4.9. Data Analysis

All experiments were performed in three biological replicates with three technical replicates in each experiment. The data were analyzed and visualized using GraphPad Prism version 6.00 for Windows (GraphPad Software, USA, www.graphpad.com). In each experiment, a comparison with a negative control was performed using the nonparametric Kruskal–Wallis test of variance. Significant differences from control were considered at *p* < 0.05.

## 5. Conclusions

Both (−)-myrtenol and (+)-myrtenol have weak antibacterial and antifungal activity while demonstrating nonstrain-specific bactericidal and fungicidal effects and exhibiting synergism with amikacin and benzalkonium chloride in relation to planktonic cells and biofilms. The mechanism of these effects appears as a consequence of the membranotropic property of the compound against bacterial and fungal cells. This may be considered as further validation that these compounds contribute to an increase in the effectiveness of various antimicrobial, antifungal, and antiseptic drugs, manifesting synergy with these compounds. Moreover, our findings confirm that terpene derivatives increase the effectiveness of benzalkonium chloride against microorganisms in the mixed community of *S. aureus* and *C. albicans*. Thus, due to the low toxicity of terpenes, these compounds could become promising agents in the treatment of infections caused by bacteria and by fungi of the genus Candida as well as mixed fungal–bacterial infections, including resistant strains.

## Figures and Tables

**Table 1:** Minimum inhibitory concentration (MIC) and minimum bactericidal concentration (MBC) values (expressed in µg/mL) of (−)-myrtenol, (+)-myrtenol, amikacin, and benzalkonium chloride (BAC) against *S. aureus* isolates.

Strains	(−)-Myrtenol		(+)-Myrtenol		Amikacin		BAC	
	MIC	MBC	MIC	MBC	MIC	MBC	MIC	MBC
*S. aureus* ATCC 29213 (MSSA)	1024	1024	512	512	4	8	0.5	1
*S. aureus* 18 (MSSA)	1024	1024	1024	1024	16	32	0.25	1
*S. aureus* 25 (MSSA)	1024	1024	1024	1024	8	16	0.25	0.5
*S. aureus* 26 (MSSA)	1024	1024	512	512	16	16	0.5	1
*S. aureus* 27 (MSSA)	1024	1024	512	512	4	16	0.5	1
*S. aureus* 1053 (MRSA)	1024	1024	2048	2048	128	512	0.5	2
*S. aureus* 1065 (MRSA)	1024	1024	512	512	128	256	0.5	2
*S. aureus* 1130 (MRSA)	512	1024	512	512	256	512	0.5	2
*S. aureus* 1145 (MRSA)	1024	512	512	512	64	1024	1	1
*S. aureus* 1167 (MRSA)	2048	2048	512	512	256	512	0.5	2
*S. aureus* 1168 (MRSA)	2048	2048	512	512	8	8	0.5	1
*S. aureus* 1173 (MRSA)	512	512	256	256	256	256	0.5	1

**Table 2:** Minimum inhibitory concentration (MIC) and minimum fungicidal concentration (MFC) (expressed in µg/mL) of (−)-myrtenol, (+)-myrtenol, fluconazole and benzalkonium chloride (BAC) against *C. albicans* isolates.

Strains	(−)-Myrtenol		(+)-Myrtenol		Fluconazole		BAC	
	MIC	MFC	MIC	MFC	MIC	MFC	MIC	MFC
*C. albicans* 722	2048	2048	1024	1024	8	8	0.5	2
*C. albicans* 761	1024	1024	2048	2048	8	8	0.5	1
*C. albicans* 661 ^FR^	1024	1024	2048	2048	512	512	0.5	1
*C. albicans* 672 ^FR^	1024	1024	2048	2048	512	512	0.5	0.5
*C. albicans* 688 ^FR^	1024	1024	2048	2048	512	512	0.5	1
*C. albicans* 701 ^FR^	2048	2048	2048	2048	512	512	1	1
*C. albicans* 703 ^FR^	1024	1024	1024	1024	512	512	0.5	2
*C. albicans* 748 ^FR^	1024	1024	2048	2048	512	512	1	2
*C. albicans* 762 ^FR^	1024	1024	2048	2048	512	512	1	2
*C. albicans* 763 ^FR^	2048	2048	2048	2048	512	512	0.5	1

**Table 3:** FICI values of amikacin and benzalkonium chloride in combination with either (−)-myrtenol or (+)-myrtenol against various isolates of *S. aureus*.

Strains	Amikacin				Benzalkonium Chloride			
	Growth Repression		Biofilm Prevention		Growth Repression		Biofilm Prevention	
	(−)-Myrtenol	(+)-Myrtenol	(−)-Myrtenol	(+)-Myrtenol	(−)-Myrtenol	(+)-Myrtenol	(−)-Myrtenol	(+)-Myrtenol
*S. aureus* ATCC (MSSA)	0.30	0.50	0.38	1.00	1.25	0.75	0.75	1.50
*S. aureus* 18 (MSSA)	0.50	0.31	1.12	0.75	0.75	0.5	0.63	0.75
*S. aureus* 25 (MSSA)	0.75	0.31	1.00	0.50	2.25	0.5	1.50	0.75
*S. aureus* 26 (MSSA)	0.75	0.75	0.75	1.25	0.75	0.75	0.75	0.28
*S. aureus* 27 (MSSA)	0.38	0.50	0.75	1.00	0.75	0.75	0.50	1.50
*S. aureus* 1053 (MRSA)	0.75	0.38	0.38	0.38	0.75	0.5	0.63	0.38
*S. aureus* 1065 (MRSA)	0.75	0.50	1.12	1.25	0.75	1.25	0.19	2.25
*S. aureus* 1130 (MRSA)	0.75	0.38	1.00	1.00	0.75	0.75	1.00	1.00
*S. aureus* 1145 (MRSA)	0.75	0.50	0.25	0.63	1.25	0.5	0.16	0.63
*S. aureus* 1167 (MRSA)	0.31	0.75	0.31	0.31	0.5	1.25	0.38	0.75
*S. aureus* 1168 (MRSA)	0.38	0.31	0.28	0.31	0.75	1.25	0.38	1.25
*S. aureus* 1173 (MRSA)	0.75	0.75	0.625	1.50	1.25	1.25	0.16	0.53
Fraction of strains with shown synergy	42%	75%	42%	33%	8%	33%	50%	17%

**Table 4:** FICI values of fluconazole and benzalkonium chloride in combination with either (−)-myrtenol or (+)-myrtenol against various isolates of *C. albicans*.

Strains	Fluconazole				Benzalkonium Chloride			
	Growth Repression		Biofilm Prevention		Growth Repression		Biofilm Prevention	
	(−)-Myrtenol	(+)-Myrtenol	(−)-Myrtenol	(+)-Myrtenol	(−)-Myrtenol	(+)-Myrtenol	(−)-Myrtenol	(+)-Myrtenol
*C. albicans* 722	1.25	1.25	0.37	0.40	0.50	0.50	1.25	0.31
*C. albicans* 761	1.25	1.25	0.37	0.50	0.75	0.50	0.38	0.50
*C. albicans* 661 ^FR^	1.25	0.75	1.25	1.25	0.75	0.38	1.25	0.50
*C. albicans* 672 ^FR^	0.27	0.50	0.28	0.50	0.75	0.50	0.38	0.75
*C. albicans* 688 ^FR^	1.25	0.75	0.26	1.25	0.50	0.50	0.75	0.75
*C. albicans* 701 ^FR^	0.28	0.27	0.75	0.75	0.75	0.75	0.50	0.38
*C. albicans* 703 ^FR^	0.38	0.27	0.31	4.25	0.75	0.50	0.75	0.75
*C. albicans* 748 ^FR^	1.25	0.27	0.26	1.25	0.75	0.50	0.75	0.50
*C. albicans* 762 ^FR^	1.25	0.27	1.25	1.25	0.38	0.50	0.50	0.38
*C. albicans* 763 ^FR^	0.27	0.50	0.75	0.30	0.50	1.25	0.75	0.50
Fraction of strains with shown synergy	36%	64%	54%	36%	45%	81%	36%	72%

## Data Availability

All data are included in the manuscript.

## Supplementary Materials

The following supporting information can be downloaded at: https://www.mdpi.com/article/10.3390/antibiotics11121743/s1, Table S1. MIC, FIC, and FICI values of amikacin in combination with either (−)-myrtenol or (+)-myrtenol (expressed in µg/mL) against various isolates of S. aureus. Table S2. Biofilm preventing concentrations (BPC), fractional biofilm preventing concentrations, and FICI values of amikacin in combination with either (−)-myrtenol or (+)-myrtenol (expressed in µg/mL) against various isolates of *S. aureus*. Table S3. MIC, FIC, and FICI values of benzalkonium chloride (BAC) in combination with either (−)-myrtenol or (+)-myrtenol (expressed in µg/mL) against various isolates of *S. aureus*. Table S4. Biofilm preventing concentrations (BPC), fractional biofilm preventing concentrations, and FICI values of benzalkonium chloride (BAC) in combination with either (−)-myrtenol or (+)myrtenol (expressed in µg/mL) against various isolates of *S. aureus*. Table S5. MIC, FIC, and FICI values of fluconazole in combination with either (−)-myrtenol or (+)-myrtenol (expressed in µg/mL) against various isolates of *C. albicans*. Table S6. Biofilm preventing concentrations (BPC), fractional biofilm preventing concentrations, and FICI values of fluconazole in combination with either (−)-myrtenol or (+)-myrtenol (expressed in µg/mL) against various isolates of *C. albicans*. Table S7. MIC, FIC, and FICI values of benzalkonium chloride (BAC) in combination with either (−)-myrtenol or (+)-myrtenol (expressed in µg/mL) against various isolates of *C. albicans*. Table S8. Biofilm preventing concentrations (BPC), fractional biofilm preventing concentrations, and FICI values of benzalkonium chloride (BAC) in combination with either (−)-myrtenol or (+)-myrtenol (expressed in µg/mL) against various isolates of *C. albicans*.
