# Supplementary material for: Increasing the Efficacy of Treatment of Staphylococcus aureus–Candida albicans Mixed Infections with Myrtenol
Source: Antibiotics (Basel). 2022 Dec 2;11(12):1743. doi: 10.3390/antibiotics11121743 (PMC9774912; doi:10.3390/antibiotics11121743)
Supplement: Supplementary file 1 [file antibiotics-11-01743-s001.zip › antibiotics-2071664-supplementary.pdf]

antibiotics

MDPI

Supplementary Materials

Table S1. MIC, FIC and FICI values of Amikacin in combination with either (-)-Myrtenol or (+)-Myrtenol (expressed in µg/mL) against various isolates of *S. aureus*.

Strains	MIC		FIC		FICI	MIC	FIC		FICI
	Amk	(-)-Myrtenol	Amk	(-)-Myrtenol	(-)-Myrtenol	(+)-Myrtenol	Amk	(+)-Myrtenol	(+)-Myrtenol
S. aureus ATCC	8	1024	0.5	256	0.30	512	2	128	0.50
S. aureus 18	32	1024	8	256	0.50	1024	2	256	0.31
S. aureus 25	16	1024	8	256	0.75	1024	1	256	0.31
S. aureus 26	16	1024	8	256	0.75	512	8	128	0.75
S. aureus 27	16	1024	2	256	0.38	512	8	128	0.50
S. aureus 1053	512	1024	256	256	0.75	2048	128	512	0.38
S. aureus 1065	256	1024	128	256	0.75	512	64	128	0.50
S. aureus 1130	512	1024	256	256	0.75	512	128	128	0.38
S. aureus 1145	1024	512	512	128	0.75	512	256	128	0.50
S. aureus 1167	512	2048	32	512	0.31	512	256	128	0.75
S. aureus 1168	8	2048	1	512	0.38	512	1	128	0.31
S. aureus 1173	256	512	128	128	0.75	256	256	64	0.75

Table S2. Biofilm preventing concentrations (BPC), fractional biofilm preventing concentrations (FIC) and FICI values of Amikacin in combination with either (-)-Myrtenol or (+)-Myrtenol (expressed in µg/mL) against various isolates of *S. aureus*.

Strains	BPC		FIC		FICI	BPC	FIC		FICI
	Amk	(-)-Myrtenol	Amk	(-)-Myrtenol	(-)-Myrtenol	(+)-Myrtenol	Amk	(+)-Myrtenol	(+)-Myrtenol
S. aureus ATCC	4	1024	0.5	256	0.38	512	2	256	1.00
S. aureus 18	64	2048	64	256	1.12	1024	32	256	0.75
S. aureus 25	64	512	32	256	1.00	512	2	256	0.50
S. aureus 26	64	1024	32	256	0.75	1024	16	256	1.25
S. aureus 27	32	1024	16	256	0.75	512	8	256	1.00
S. aureus 1053	1024	2048	256	256	0.38	2048	256	256	0.38
S. aureus 1065	1024	2048	1024	256	1.12	1024	1024	256	1.25
S. aureus 1130	128	512	64	256	1.00	512	512	256	1.00
S. aureus 1145	1024	1024	128	128	0.25	1024	512	128	0.63
S. aureus 1167	512	2048	32	512	0.31	2048	128	512	0.31
S. aureus 1168	32	2048	1	512	0.28	2048	1	512	0.31
S. aureus 1173	256	1024	128	128	0.625	256	512	128	1.50

Table S3. MIC, FIC and FICI values of Benzalkonium Chloride (BAC) in combination with either (-)-Myrtenol or (+)-Myrtenol (expressed in μg/mL) against various isolates of *S. aureus*.

Strains	MIC		FIC		FICI	MIC	FIC		FICI
	BAC	(-)-Myrtenol	BAC	(-)-Myrtenol	(-)-Myrtenol	(+)-Myrtenol	BAC	(+)-Myrtenol	(+)-Myrtenol
S. aureus ATCC	1	1024	1	256	1.25	512	1	128	0.75
S. aureus 18	1	1024	0.5	256	0.75	1024	0.25	256	0.5
S. aureus 25	0.5	1024	1	256	2.25	1024	0.25	256	0.5
S. aureus 26	1	1024	0.5	256	0.75	512	1	128	0.75
S. aureus 27	1	1024	0.5	256	0.75	512	1	128	0.75
S. aureus 1053	2	1024	1	256	0.75	2048	0.5	512	0.5
S. aureus 1065	2	1024	1	256	0.75	512	2	128	1.25
S. aureus 1130	2	1024	1	256	0.75	512	1	128	0.75
S. aureus 1145	1	512	1	128	1.25	512	0.5	128	0.5
S. aureus 1167	2	2048	0.5	512	0.5	512	1	128	1.25
S. aureus 1168	1	2048	0.5	512	0.75	512	1	128	1.25
S. aureus 1173	1	512	1	128	1.25	256	1	64	1.25

Table S4. Biofilm preventing concentrations (BPC), fractional biofilm preventing concentrations (FIC) and FICI values of Benzalkonium Chloride (BAC) in combination with either (-)-Myrtenol or (+)-Myrtenol (expressed in μg/mL) against various isolates of *S. aureus*.

Strains	BPC		FIC		FICI	BPC	FIC		FICI
	BAC	(-)-Myrtenol	BAC	(-)-Myrtenol	(-)-Myrtenol	(+)-Myrtenol	BAC	(+)-Myrtenol	(+)-Myrtenol
S. aureus ATCC	*1*	*1024*	*0.5*	*256*	*0.75*	*512*	*1*	*256*	*1.50*
S. aureus 18	*1*	*2048*	*0.5*	*256*	*0.63*	*1024*	*0.5*	*256*	*0.75*
S. aureus 25	*1*	*512*	*1*	*256*	*1.50*	*512*	*0.25*	*256*	*0.75*
S. aureus 26	*1*	*1024*	*0.5*	*256*	*0.75*	*1024*	*0.03*	*256*	*0.28*
S. aureus 27	*1*	*1024*	*0.25*	*256*	*0.50*	*512*	*1*	*256*	*1.50*
S. aureus 1053	*2*	*2048*	*1*	*256*	*0.63*	*2048*	*0.5*	*256*	*0.38*
S. aureus 1065	*1*	*2048*	*0.06*	*256*	*0.19*	*1024*	*2*	*256*	*2.25*
S. aureus 1130	*2*	*512*	*1*	*256*	*1.00*	*512*	*1*	*256*	*1.00*
S. aureus 1145	*2*	*1024*	*0.06*	*128*	*0.16*	*1024*	*1*	*128*	*0.63*
S. aureus 1167	*2*	*2048*	*0.25*	*512*	*0.38*	*2048*	*1*	*512*	*0.75*
S. aureus 1168	*1*	*2048*	*0.12*	*512*	*0.38*	*2048*	*1*	*512*	*1.25*
S. aureus 1173	*1*	*1024*	*0.03*	*128*	*0.16*	*256*	*0.03*	*128*	*0.53*

Table S5. MIC, FIC and FICI values of Fluconazole in combination with either (-)-Myrtenol or (+)-Myrtenol (expressed in μg/mL) against various isolates of *C. albicans*.

strains	MIC		FIC		FICI	MIC	FIC		FICI
	Flu	(-)-Myrtenol	Flu	(-)-Myrtenol	(-)-Myrtenol	(+)-Myrtenol	Flu	(+)-Myrtenol	(+)-Myrtenol
C. albicans ATCC	512	1024	512	256	1.25	1024	8	256	0.27
C. albicans 661	512	1024	512	256	1.25	2048	256	512	0.75
C. albicans 672	512	1024	8	256	0.27	2048	8	512	0.50
C. albicans 688	512	1024	512	256	1.25	2048	256	512	0.75
C. albicans 701	512	2048	16	512	0.28	2048	8	512	0.27
C. albicans 703	64	1024	8	256	0.38	1024	8	256	0.27
C. albicans 722	8	2048	8	512	1.25	1024	8	256	1.25
C. albicans 748	512	1024	512	256	1.25	2048	8	512	0.27
C. albicans 761	8	1024	8	256	1.25	2048	8	512	1.25
C. albicans 762	512	1024	512	256	1.25	2048	8	512	0.27
C. albicans 763	512	2048	8	512	0.27	2048	128	512	0.50

Table S6. Biofilm preventing concentrations (BPC), fractional biofilm preventing concentrations (FIC) and FICI values of Fluconazole in combination with either (-)-Myrtenol or (+)-Myrtenol (expressed in μg/mL) against various isolates of *C. albicans*.

Strains	BPC		FIC		FICI	BPC	FIC		FICI
	Flu	(-)-Myrtenol	Flu	(-)-Myrtenol	(-)-Myrtenol	(+)-Myrtenol	Flu	(+)-Myrtenol	(+)-Myrtenol
C. albicans ATCC	512	1024	512	256	1.25	1024	64	256	0.75
C. albicans 661	512	1024	512	256	1.25	2048	512	512	1.25
C. albicans 672	512	1024	16	256	0.28	2048	8	512	0.50
C. albicans 688	512	1024	8	256	0.26	2048	512	512	1.25
C. albicans 701	512	2048	256	512	0.75	2048	256	512	0.75
C. albicans 703	128	1024	8	256	0.31	1024	32	256	4.25
C. albicans 722	64	2048	8	512	0.37	1024	64	256	0.40
C. albicans 748	512	1024	8	256	0.26	2048	512	512	1.25
C. albicans 761	512	1024	64	256	0.37	2048	128	512	0.50
C. albicans 762	512	1024	512	256	1.25	2048	512	512	1.25
C. albicans 763	512	2048	256	512	0.75	2048	8	512	0.30

Table S7. MIC, FIC and FICI values of Benzalkonium Chloride (BAC) in combination with either (-)-Myrtenol or (+)-Myrtenol (expressed in μg/mL) against various isolates of *C. albicans*.

Strains	MIC		FIC		FICI	MIC	FIC		FICI
	BAC	(-)-Myrtenol	BAC	(-)-Myrtenol	(-)-Myrtenol	(+)-Myrtenol	BAC	(+)-Myrtenol	(+)-Myrtenol
C. albicans ATCC	1	1024	0.125	256	0.38	1024	0.125	256	0.50
C. albicans 661	0.5	1024	0.25	256	0.75	2048	0.125	512	0.38
C. albicans 672	0.5	1024	0.25	256	0.75	2048	0.125	512	0.50
C. albicans 688	0.5	1024	0.125	256	0.50	2048	0.25	512	0.50
C. albicans 701	1	2048	0.5	512	0.75	2048	0.25	512	0.75
C. albicans 703	0.5	1024	0.25	256	0.75	1024	0.25	256	0.50
C. albicans 722	0.5	2048	0.125	512	0.50	1024	0.125	256	0.50
C. albicans 748	1	1024	0.5	256	0.75	2048	0.25	512	0.50
C. albicans 761	0.5	1024	0.25	256	0.75	2048	0.125	512	0.50
C. albicans 762	1	1024	0.125	256	0.38	2048	0.25	512	0.50
C. albicans 763	0.5	2048	0.125	512	0.50	2048	0.25	512	1.25

Table S8. Biofilm preventing concentrations (BPC), fractional biofilm preventing concentrations (FIC) and FICI values of Benzalkonium Chloride (BAC) in combination with either (-)-Myrtenol or (+)-Myrtenol (expressed in μg/mL) against various isolates of *C. albicans*.

Strains	BPC		FIC		FICI	BPC	FIC		FICI
	BAC	(-)-Myrtenol	BAC	(-)-Myrtenol	(-)-Myrtenol	(+)-Myrtenol	BAC	(+)-Myrtenol	(+)-Myrtenol
C. albicans ATCC	0.25	1024	0.125	256	0.75	1024	0.125	256	0.38
C. albicans 661	0.125	2048	0.125	512	1.25	1024	0.125	256	0.50
C. albicans 672	1	2048	0.125	512	0.38	1024	0.125	256	0.75
C. albicans 688	0.5	2048	0.25	512	0.75	1024	0.125	256	0.75
C. albicans 701	0.5	2048	0.125	512	0.50	2048	0.125	512	0.38
C. albicans 703	0.25	1024	0.125	256	0.75	1024	0.125	256	0.75
C. albicans 722	0.125	1024	0.125	256	1.25	2048	0.25	512	0.31
C. albicans 748	1	2048	0.5	512	0.75	1024	0.125	256	0.50
C. albicans 761	1	2048	0.125	512	0.38	1024	0.25	256	0.50
C. albicans 762	1	2048	0.25	512	0.50	1024	0.125	256	0.38
C. albicans 763	0.5	2048	0.25	512	0.75	2048	0.125	512	0.50
